# The Relationships Between Social Media and Human Papillomavirus Awareness and Knowledge: Cross-sectional Study

**DOI:** 10.2196/37274

**Published:** 2022-09-20

**Authors:** Soojung Jo, Keenan A Pituch, Nancy Howe

**Affiliations:** 1 School of Nursing Purdue University West Lafayette, IN United States; 2 Edson College of Nursing and Health Innovation Arizona State University Phoenix, AZ United States

**Keywords:** papillomavirus infections, vaccination, social media, health promotion, public reporting of health care data, human papillomavirus

## Abstract

**Background:**

Human papillomavirus (HPV) is the most common sexually transmitted infection. HPV can infect both females and males, and it can cause many cancers, including anal, cervical, vaginal, vulvar, and penile cancers. HPV vaccination rates are lower than vaccination rates within other national vaccination programs, despite its importance. Research literature indicates that people obtain health-related information from internet sources and social media; however, the association between such health-seeking behavior on social media and HPV-related behaviors has not been consistently demonstrated in the literature.

**Objective:**

This study aims to examine the association between social media usage and HPV knowledge and HPV awareness.

**Methods:**

This study analyzed public health data collected through the Health Information National Trends Survey (HINTS) conducted by the US National Cancer Institute. The analysis used data collected in 2020; in total, 2948 responses were included in the analysis. Six HPV-related questions were used to identify HPV awareness, HPV vaccine awareness, and HPV knowledge about HPV-related cancers. Four questions about social media usage and one question about online health information–seeking behavior were used to analyze the associations between social media usage and HPV-related behaviors. Initially, six logistic regressions were conducted using replicate weights. Based on the results, significant factors were included in a second set of regression analyses that also included demographic variables.

**Results:**

About half of the respondents were aware of HPV (68.40%), the HPV vaccine (64.04%), and the relationship between HPV and cervical cancer (48.00%). However, fewer respondents were knowledgeable about the relationships between HPV and penile cancer (19.18%), anal cancer (18.33%), and oral cancer (19.86%). Although social media usage is associated with HPV awareness, HPV vaccine awareness, and knowledge of cervical cancer, these associations were not significant after adjusting for demographic variables. Those less likely to report HPV awareness and knowledge included older participants, males, those with a household income of less than US $20,000, those with a formal education equal to or less than high school, or those who resided in a household where adults are not fluent in English.

**Conclusions:**

After adjusting for demographic variables, social media use was not related to HPV knowledge and awareness, and survey respondents were generally not aware that HPV can lead to specific types of cancer, other than cervical cancer. These results suggest that perhaps a lack of high-quality information on social media may impede HPV awareness and knowledge. Efforts to educate the public about HPV via social media might be improved by using techniques like storytelling or infographics, especially targeting vulnerable populations, such as older participants, males, those with low incomes, those with less formal education, or those who reside in the United States but are not fluent in English.

## Introduction

Human papillomavirus (HPV) is a common sexually transmitted infection [1]. HPV infection is associated with many different cancers, including anal, cervical, vaginal, vulvar, and penile cancers [2], and can infect both females and males [3]. About 39,221 cancers annually can be caused by HPV [4]. HPV infection is the cause of nearly all cervical cancers, about 90% of all anal cancers, about 75% of all vaginal cancers, about 70% of all vulvar cancers, and about 60% of all penile cancers [2]. Recent studies have shown strong relationships among HPV infection, tobacco use, and oropharynx cancers [5], which make up about 70% of all HPV-related cancers in the United States [2]. According to the American Cancer Society, it is estimated that 1,918,030 new cancers are diagnosed and 609,360 deaths are attributed to cancer annually. In 2022, 14,100 new cases of cervical cancer were diagnosed and 4280 deaths were attributed to cervical cancer [6].

Detection of HPV infection is difficult since most HPV infections are typically asymptomatic. HPV infection is easily preventable by vaccination. HPV vaccination can prevent up to 93% of cervical cancer occurrences [7]. Despite the importance of HPV vaccination, the rate of HPV vaccination in the United States remains low. Among adolescents aged 13 to 17 years, HPV vaccination completion in the United States was estimated at 54.2% in 2019 and 58.6% in 2020, a rate that is much lower than the vaccination rate for measles, mumps, and rubella or for tetanus, diphtheria, and pertussis, which all have vaccination rates of more than 90% [8].

HPV vaccination among adolescents is highly associated with parental desire and recommendation rather than a health care provider’s recommendation [9,10]. Literature has shown that HPV vaccination intention among parents for their children was associated with HPV awareness and HPV knowledge [11,12]. Despite the importance of HPV knowledge, research indicates that people have little knowledge about HPV [13]. Health care providers’ recommendations or information on HPV is needed to increase HPV awareness and knowledge. However, research indicates that people do not obtain information about HPV and related cancers from health care providers. Instead, HPV information is gathered from different internet sites and social media [12,14].

The internet enables people to search online for health-related information. A literature review reported that people who seek health-related information for specific diseases or for public health concerns felt emotionally supported by peer interactions that occur through social media [15]. Even though health issues are private, people who trust social media interactions are more likely to disclose their health issues over social media [16], leading to greater engagement with others about health issues and greater sharing of health-related concerns.

In terms of cancer prevention behavior, some research has shown the positive impact of social media [17,18]. People who use social media are more likely to have heard of hepatitis C virus and HPV [17]. A systematic review indicated that individuals who engage in social media have higher HPV awareness [18]. However, the results of the impact of social media were inconsistent. Social media engagement was not associated with HPV vaccine uptake [18]. While interventions using social media could improve HPV knowledge [18], it is difficult to assess whether there is an association between social media and HPV knowledge. The quality of the information available through social media mattered as well. Research showed positive encouragement for HPV vaccination on Twitter [19]. However, research that analyzed websites that mentioned HPV discovered that antivaccine content was greater than provaccine content: 50.7% of content was antivaccine and only 37.4% of content was provaccine [20]. Similarly, the majority of YouTube videos on HPV were antivaccine videos (57%), whereas only 31% were provaccine videos [21].

Antivaccine messages about HPV can negatively impact HPV awareness and HPV knowledge, and unvaccinated people are likely to contribute to an infectious disease outbreak. For example, 49 out of 110 (45%) patients infected in the California measles outbreak of 2014-2015 were unvaccinated, and 28 of them intentionally did not get vaccinated because of their health beliefs [22]. Social media may contribute to an antivaccine sentiment, as misinformation can be quickly and easily disseminated, increasing exposure to misinformation [23]. Moreover, social media communities could further spread misinformation by interacting with other community members and reinforce the users’ incorrect beliefs. In this way, the impact of misinformation could be much greater in a small, robust antivaccination community than the impact of accurate, more traditional forms of online information, such as certified web pages. Despite the important role of social media, no research has evaluated the influence of social media usage on HPV knowledge. Therefore, this study aims to examine the association between social media usage and HPV knowledge and HPV awareness.

## Methods

### Recruitment

In this study, a secondary data analysis was performed using public health data collected through the Health Information National Trends Survey (HINTS) conducted by the US National Cancer Institute. HINTS is a national survey that collects data on cancer and health-related knowledge and attitudes about cancer and preventive behaviors. This study is based on the most recent data, which was collected from the 2020 HINTS 5, Cycle 4 survey. Data sampling was based on random sampling using addresses, and the data were grouped into two stratifications: high concentrations of minority populations and low concentrations of minority populations. The survey was sent to the selected population by mail, and survey respondents returned their surveys by mail. Data collection occurred from February 24, 2020, through June 15, 2020. The survey was sent to 15,347 households by grouping two sampling strata: high concentrations of minority populations (n=11,050) and low concentrations of minority populations (n=4300). The response rate was 37%, with a total of 3865 responses collected. In this study, responses with missing data were excluded, resulting in a total of 2948 responses included in the final analysis.

### Ethical Considerations

The data are publicly available on the HINTS website, enabling anyone to use the data without requiring specific approval from an institutional review board.

### Measurements

Six HPV-related questions were used as dependent variables. HPV awareness was evaluated by the question, “Have you ever heard of HPV? HPV stands for Human Papillomavirus. It is not HCV, HIV, HSV, or herpes.” HPV vaccine awareness was evaluated by the question, “A vaccine to prevent HPV infection is available and is called the HPV shot, cervical cancer vaccine, GARDASIL. Before today, have you ever heard of the cervical cancer vaccine or HPV shot?” Data show that cervical cancer vaccination is more commonly advertised than HPV vaccination [24] and that more people are aware of cervical and HPV vaccination than are aware of HPV infection [10]. Hence, we analyzed each of two HPV and HPV vaccine awareness variables.

Four questions targeted HPV knowledge by asking about four HPV-related cancers: “Do you think HPV can cause a) cervical, b) penile, c) anal, or d) oral cancer?” Previous studies have reported that there is a lack of knowledge about the relationship between HPV and the cancers that occur in males, including penile and anal cancers. Only 36.1% of respondents in prior studies were aware that HPV can cause noncervical cancers, whereas 79.6% were aware that HPV can cause cervical cancer [25]. Therefore, this study analyzed each of the four cancers as dependent variables instead of summing a single question as a continuous variable. HPV knowledge and HPV vaccine awareness were coded as “yes” or “no.” HPV knowledge was screened by the HPV awareness question with three possible responses: “yes,” “no,” and “not sure.” In this study, respondents who answered either “no” or “not sure” about HPV knowledge were both coded as “no” with respect to the HPV knowledge questions.

One extended question addressed four different methods for using social media. These four methods of using the internet were defined in a single question as follows: “Sometimes people use the Internet to connect with other people online through social networks like Facebook or Twitter. This is often called ‘social media.’ In the last 12 months, have you used the Internet for any of the following reasons? a) To visit a social networking site, such as Facebook or LinkedIn. b) To share health information on social networking sites, such as Facebook or Twitter. c) To participate in an online forum or support group for people with a similar health or medical issue. d) To watch a health-related video on YouTube.” Each of the four possibilities was considered as one variable. All four variables were coded as either “yes” or “no.” In addition to the social media questions, online health information–seeking behavior was measured by asking, “In the past 12 months, have you used a computer, smartphone, or other electronic means to look for health or medical information for yourself?” Answers were coded as “yes” or “no.”

Control variables included demographic characteristics, English proficiency, and descriptions of online health information–seeking behaviors. Demographic characteristics included age, biological sex, household income (“<US $20,000,” “$20,000 to <$35,000,” “$35,000 to <$50,000,” “$50,000 to <$75,000,” or “≥$75,000”), educational level (“equal to or less than high school,” “post–high school training to college graduate,” or “postgraduate”), employment (“employed” or “other”), and marital status (“single, never been married” or “other”). The HINTS survey gathered data on linguistically isolated strata by identifying that 30% of households do not include members older than 14 years who speak English well. English proficiency is a significant factor associated with HPV knowledge, especially among immigrant populations [26]. Therefore, English proficiency was included as a control variable and was coded as “yes” or “no.”

### Statistical Analysis

Initially, six logistic regressions were used to examine the relationship between social media usage and HPV-related behaviors: HPV awareness, HPV vaccine awareness, and four HPV knowledge variables. Based on the results of the analysis, associations between three HPV-related variables and the significant predictors were further analyzed by adding demographic characteristics as control variables. Replicate weights using the jackknife replication method were used to estimate the sampling variability among the population estimates. To strike a reasonable balance between type I and type II error rates, we used an α of .01 when testing each regression coefficient and obtained the corresponding 99% CI for each odds ratio (OR). All analyses were conducted using SAS software (version 9.4; SAS Institute Inc).

## Results

### Demographic Characteristics

Table 1 shows the demographic characteristics of the sample. The mean age was 46.70 (SE 0.33) years. About half of the respondents were female (50.47%—weighted proportions are reported throughout the paper) and earned US $75,000 or more in household income (44.82%). Most respondents had post–high school or some college training or were college graduates (60.01%), followed by those with an educational level equal to or less than high school (27.61%) and postgraduates (12.38%). About one-third were employed (36.87%). There were 32.25% respondents who were single and had never been married. In total, 7.31% respondents indicated that their households did not include adults who are fluent in English.

**Table 1:** Demographic characteristics of the sample.

Characteristic			Sample (N=2948), n (%)		Weighted sample, n		Weighted proportion, % (SE)
**Sex**							
	Female	1691 (57.36)		104,697,568		50.47 (0.56)	
	Male	1257 (42.64)		102,732,540		49.53 (0.56)	
**Household income (US $)**							
	<20,000	441 (14.96)		27,599,477		13.31 (0.97)	
	20,000 to <35,000	372 (12.62)		22,584,273		10.89 (0.81)	
	35,000 to <50,000	375 (12.72)		24,560,620		11.84 (0.90)	
	50,000 to <75,000	535 (18.15)		39,722,602		19.15 (1.63)	
	≥75,000	1225 (41.55)		92,963,137		44.82 (1.73)	
**Educational level**							
	Equal to or less than high school	662 (22.46)		57,275,441		27.61 (1.04)	
	Post–high school training, some college, or college graduate	1696 (57.53)		124,479,334		60.01 (1.23)	
	Postgraduate	590 (20.01)		25,675,333		12.38 (0.75)	
**Employment**							
	Employed	1333 (45.22)		76,489,111		36.87 (1.27)	
	Other^a^	1615 (54.78)		130,940,997		63.13 (1.27)	
**Marital status**							
	Single, never been married	528 (17.91)		66,886,401		32.25 (0.51)	
	Other^b^	2420 (82.09)		140,543,707		67.75 (0.51)	
**English proficiency**							
	Good	2689 (91.21)		192,276,056		92.69 (0.57)	
	Not good	259 (8.79)		15,154,053		7.31 (0.57)	
**Online health information seeking**							
	Yes	2204 (74.76)		156,874,229		75.63 (1.34)	
	No	744 (25.24)		50,555,880		24.37 (1.34)	
**Visited a social networking site, such as Facebook or LinkedIn**							
	Yes	2096 (71.10)		160,485,232		77.37 (1.17)	
	No	852 (28.90)		46,944,877		22.63 (1.17)	
**Shared health information on social networking sites, such as Facebook or Twitter**							
	Yes	416 (14.11)		31,485,688		15.18 (1.05)	
	No	2532 (85.89)		175,944,421		84.82 (1.05)	
**Participated in an online forum or support group for people with a similar health or medical issue**							
	Yes	268 (9.09)		20,857,999		10.06 (0.78)	
	No	2680 (90.91)		186,572,110		89.94 (0.78)	
**Watched a health-related video on YouTube**							
	Yes	1156 (39.21)		87,106,159		41.99 (1.40)	
	No	1792 (60.79)		120,323,949		58.01 (1.40)	
**HPV^c^ awareness**							
	Yes	1953 (66.25)		141,878,406		68.40 (1.60)	
	No	995 (33.75)		65,551,702		31.60 (1.60)	
**HPV vaccine awareness**							
	Yes	1855 (62.92)		132,835,764		64.04 (1.45)	
	No	1093 (37.08)		74,594,344		35.96 (1.45)	
**HPV knowledge (can cause cervical cancer)**							
	Yes	1412 (47.90)		99,569,406		48.00 (1.55)	
	No	1536 (52.10)		107,860,702		52.00 (1.55)	
**HPV knowledge (can cause penile cancer)**							
	Yes	554 (18.79)		39,791,723		19.18 (1.26)	
	No	2394 (81.21)		167,638,385		80.82 (1.26)	
**HPV knowledge (can cause anal cancer)**							
	Yes	531 (18.01)		38,012,201		18.33 (1.34)	
	No	2417 (81.99)		169,417,907		81.67 (1.34)	
**HPV knowledge (can cause oral cancer)**							
	Yes	566 (19.20)		41,193,057		19.86 (1.31)	
	No	2382 (80.80)		166,237,051		80.14 (1.31)	

^a^Unemployed, homemaker, student, retired, disabled, or other response.

^b^Married, living as married, or living with a romantic partner; divorced; widowed; or separated.

^c^HPV: human papillomavirus.

With respect to social media usage–related variables, about 77.37% of respondents have visited a social networking site, 15.18% have shared health information on social networking sites, 10.06% have participated in an online forum or support group for people with similar health or medical issues, and 41.99% have watched a health-related video on YouTube. About 75.63% of respondents indicated that they seek health information online.

More than half of the respondents were aware of HPV (68.40%), and a similar number were aware of the HPV vaccine (64.04%). Less than half of the respondents knew that HPV could cause cervical cancer (48.00%). However, far fewer respondents were knowledgeable about the relationships between HPV and penile cancer (19.18%), HPV and anal cancer (18.33%), and HPV and oral cancer (19.86%).

### Relationship Between Social Media Usage and HPV-Related Behaviors

Table 2 shows the results of six logistic regressions assessing the relationship between social media usage and HPV-related behaviors. In general, seeking health information online and having visited a social networking site were associated with HPV-related behaviors. Specifically, people who sought health information online were more likely to be aware of HPV (OR 2.25, 99% CI 1.49-3.40), the HPV vaccine (OR 1.85, 99% CI 1.27-2.70), and the relationship between HPV and cervical cancer (OR 2.73, 99% CI 1.69-4.42). Likewise, people who visited a social networking site were more likely to be aware of HPV (OR 2.10, 99% CI 1.28-3.43), the HPV vaccine (OR 2.10, 99% CI 1.34-3.30), and the relationship between HPV and cervical cancer (OR 1.94, 99% CI 1.17-3.22). Moreover, people who have participated in an online forum or support group for people with similar health or medical issues had higher HPV awareness (OR 2.35, 99% CI 1.05-5.26). However, having shared health information on social networking sites and having watched a health-related video on YouTube were not significant factors. Moreover, none of the social media variables were significantly related to the knowledge that HPV causes penile cancer, anal cancer, or oral cancer.

**Table 2:** Relationship between social media usage and HPV-related behaviors.

Predictor	HPV^a^ awareness			HPV vaccine awareness			HPV knowledge (can cause cervical cancer)			HPV knowledge (can cause penile cancer)			HPV knowledge (can cause anal cancer)			HPV knowledge (can cause oral cancer)	
	OR^b^ (99% CI)	*P* value	OR (99% CI)		*P* value	OR (99% CI)		*P* value	OR (99% CI)		*P* value	OR (99% CI)		*P* value	OR (99% CI)		*P* value
Online health information seeking	2.25 (1.49-3.40)	<.001	1.85 (1.27-2.70)		<.001	2.73 (1.69-4.42)		<.001	1.37 (0.64-2.91)		.27	1.40 (0.64-3.05)		.25	1.58 (0.71-3.51)		.13
Visited a social networking site, such as Facebook or LinkedIn	2.10 (1.28-3.43)	<.001	2.10 (1.34-3.30)		<.001	1.94 (1.17-3.22)		<.001	1.58 (0.82-3.03)		.07	1.22 (0.62-2.41)		.44	1.22 (0.68-2.21)		.37
Shared health information on social networking sites, such as Facebook or Twitter	1.02 (0.58-1.78)	.94	1.50 (0.92-2.45)		.03	1.17 (0.75-1.83)		.35	1.22 (0.73-2.05)		.31	1.43 (0.83-2.47)		.09	0.95 (0.54-1.68)		.82
Participated in an online forum or support group for people with a similar health or medical issue	2.35 (1.05-5.26)	.006	2.02 (0.97-4.20)		.01	1.62 (0.92-2.85)		.03	1.38 (0.74-2.59)		.17	1.10 (0.59-2.05)		.69	1.04 (0.50-2.17)		.88
Watched a health-related video on YouTube	1.10 (0.71-1.71)	.57	1.20 (0.81-1.78)		.22	1.05 (0.68-1.61)		.78	0.99 (0.58-1.67)		.94	1.06 (0.64-1.76)		.78	1.09 (0.71-1.67)		.59

^a^HPV: human papillomavirus.

^b^OR: odds ratio.

### Adjusted Associations Between Social Media Usage and HPV Outcomes

Based on the previous results, demographic variables, along with the three significant social media variables, were included in regression models for HPV awareness, HPV vaccine awareness, and knowledge that HPV can cause cervical cancer. Table 3 shows the results of these logistic regressions. Unlike the previous results from Table 2, social media usage was not significantly associated with any of the HPV variables. However, seeking health information online was marginally associated with HPV awareness (OR 1.53, 99% CI 0.99-2.39; *P*=.01). Also, knowledge of the relationship between HPV and cervical cancer (OR 1.65, 99% CI 1.00-2.74; *P*=.01) and having visited a social networking site were marginally related to HPV vaccine awareness (OR 1.62, 99% CI 0.99-2.66; *P*=.01).

Among the demographic variables, age (OR 0.97, 99% CI 0.96-0.99), sex (OR 0.47, 99% CI 0.29-0.76), income, educational level, and English proficiency were significantly associated with HPV outcomes. Older people and males were less likely to be aware of the HPV vaccine. Individuals with a household income greater than or equal to US $75,000 were more likely to be aware of the HPV vaccine compared to individuals with a household income less than US $20,000 (OR 1.97, 99% CI 1.06-3.68). Respondents who are college graduates were more likely to be aware of HPV (OR 1.79, 99% CI 1.11-2.88) and the HPV vaccine (OR 2.30, 99% CI 1.43-3.72) as well as to know about relationships between HPV and cervical cancer (OR 2.97, 99% CI 1.96-4.49) compared to respondents whose educational level did not exceed high school. Similarly, respondents who indicated that they are postgraduates were more likely to be aware of HPV (OR 2.67, 99% CI 1.21-5.91), the HPV vaccine (OR 2.76, 99% CI 1.46-5.21), and the relationship between HPV and cervical cancer (OR 5.98, 99% CI 3.40-10.50) compared to respondents whose educational level did not exceed high school. Respondents whose households included adults who are fluent in English were more likely to be aware of the HPV vaccine (OR 2.12, 99% CI 1.04-4.34).

**Table 3:** Associations between social media usage and HPV awareness, HPV vaccine awareness, and HPV knowledge.

Predictor		HPV^a^ awareness		HPV vaccine awareness			HPV knowledge (can cause cervical cancer)	
		OR^b^ (99% CI)	*P* value	OR (99% CI)	*P* value	OR (99% CI)		*P* value
Age		0.97 (0.96-0.99)	<.001	0.98 (0.96-1.00)	.002	0.98 (0.96-0.99)		<.001
Gender: male (reference: female)		0.47 (0.29-0.76)	<.001	0.30 (0.20-0.44)	<.001	0.44 (0.28-0.69)		<.001
**Income (US $)**								
	20,000 to <35,000 (reference: <20,000)	0.95 (0.48-1.87)	.83	0.91 (0.47-1.76)	.71	0.67 (0.31-1.47)		.18
	35,000 to <50,000	1.12 (0.53-2.37)	.68	1.07 (0.58-1.95)	.78	0.83 (0.39-1.78)		.52
	50,000 to <75,000	0.96 (0.42-2.16)	.88	0.73 (0.42-1.29)	.14	0.77 (0.37-1.61)		.34
	≥75,000	1.71 (0.75-3.90)	.09	1.97 (1.06-3.68)	.005	1.38 (0.62-3.05)		.29
**Educational level**								
	Post–high school training, some college, or college graduate (reference: equal to or less than high school)	1.79 (1.11-2.88)	.002	2.30 (1.43-3.72)	<.001	2.97 (1.96-4.49)		<.001
	Postgraduate	2.67 (1.21-5.91)	.002	2.76 (1.46-5.21)	<.001	5.98 (3.40-10.50)		<.001
Employed (reference: other)		0.96 (0.60-1.53)	.81	1.06 (0.67-1.69)	.72	1.05 (0.67-1.63)		.78
Single, never been married (reference: other)		1.10 (0.63-1.91)	.65	1.15 (0.67-1.99)	.49	1.19 (0.70-2.03)		.39
English proficiency		1.39 (0.72-2.68)	.19	2.12 (1.04-4.34)	.007	1.12 (0.60-2.10)		.63
Online health information seeking		1.53 (0.99-2.39)	.01	1.15 (0.74-1.79)	.40	1.65 (1.00-2.74)		.01
Visited a social networking site, such as Facebook or LinkedIn		1.44 (0.89-2.32)	.05	1.62 (0.99-2.66)	.01	1.45 (0.84-2.50)		.07
Participated in an online forum or support group for people with a similar health or medical issue		2.14 (0.95-4.82)	.02	2.11 (0.97-4.61)	.01	1.42 (0.81-2.50)		.10

^a^HPV: human papillomavirus.

^b^OR: odds ratio.

## Discussion

### Principal Findings

This study analyzed the relationship between social media usage and awareness of HPV, the HPV vaccine, and HPV-related knowledge about cervical, anal, penile, and oral cancers. Although social media usage is associated with HPV awareness and knowledge, these associations were not significant after adjusting for demographic variables and were only marginally related to HPV-related behaviors. Meanwhile, the demographic variables age, sex, educational level, income, and English proficiency were significantly associated with HPV-related behaviors.

The nonsignificant associations between social media usage and HPV awareness, HPV vaccine awareness, and knowledge related to cervical cancer might be related to the quality of information on social media. Earlier research that analyzed websites that mention HPV reported that only 4.81% of those websites included information that HPV can cause cervical cancer [20]. In addition, research that specifically analyzed Twitter postings reported that most tweets about HPV were written by nonprofessionals. Twitter tweets about HPV more often contained links to layperson blogs compared to links to professional information or websites [19]. Thus, our results showing that respondents who used social media did not possess more HPV knowledge than respondents who did not use social media may reflect the poor quality of information posted on social media and on some websites and blogs that are linked to poor-quality social media posts. Moreover, searching for health information on social media may be triggered by the needs of people who have special health concerns or health issues [15]. Using social media for personal health knowledge could not be addressed directly with respect to HPV. People may search for other topics or general health concerns when they use social media [27]. Since HPV infection does not result in any symptoms and can cause cancer multiple years after infection, people might not search specifically for information about HPV. People are less likely to search for information about HPV unless someone actually recommends that they should research HPV.

Another explanation could be the characteristics of social media for the information exchange perspectives. Using social media could limit the information that circulates within the community and could lead to a lack of knowledge [23]. People obtain health information by interacting with peers inside social media or inside specific, smaller, and more robust communities found on social media [15]. Information provided by social media could be reinforced by exposure based on the number of users and networks, so it could increase the proliferation of misinformation and could reinforce the incorrect beliefs of the viewers [23]. If there is no one to correct misinformation, people using social media may have difficulty discerning correct from incorrect information. Unfortunately, experts or government agencies are often unable to correct misinformation. Compelling personal stories that contain misinformation can be especially difficult to correct, further impeding the promotion of accurate health information on social media.

Our results also show that watching health-related videos on YouTube is not associated with either HPV awareness or HPV knowledge. We suggest that there may be three possible explanations for this unexpected result. First, the majority of YouTube videos about HPV were videos that contained an antivaccine bias: 57% of YouTube videos presented an antivaccine philosophy compared to only 31% of YouTube videos that promoted the health benefits of HPV vaccination [21]. Second, most of the viewers’ top comments about HPV-related videos highlighted potential negative side effects of vaccination and supported conspiracy theories about recommendations for HPV vaccination [21]. This combination of inaccurate and biased information and the prominence of negative viewer comments about HPV vaccination might explain why watching health-related videos on YouTube is not associated with respondents having more HPV knowledge compared to respondents who do not watch YouTube for health-related information. Finally, a third explanation is related to the way in which viewers find information on YouTube. YouTube provides personalized videos based on the viewer’s history of watching [28]. YouTube algorithms employ user-provided performance, watch history, and recognition of the specific videos that users watch to suggest additional videos to users. The nonsignificant association between HPV awareness and watching YouTube videos might be a consequence of how users find information on YouTube. People who are unaware of HPV may be less likely to enter HPV-related keywords and, therefore, their searches would be less likely to trigger YouTube algorithms to suggest videos about HPV. The HINTS questions did not ask respondents about the specific health issues that they researched online or through social media. Further research is suggested to investigate the causal relationships.

Literature indicates that the HPV vaccine is often described as a cervical cancer vaccine [24] and that respondents report greater awareness of a cervical cancer vaccine instead of its product name, GARDASIL 9, or the HPV vaccine itself [10]. There is less knowledge that HPV infection can cause cancers other than cervical cancer, including penile and oral cancers [13]. This suggests that respondents who searched online for HPV-related health information were already more knowledgeable about HPV than the respondents who did not search online for HPV-related health information. Perhaps people who have heard of HPV search online for the detailed information, and this might explain the marginal effect of seeking health information online and visiting social networking sites regarding the HPV vaccine and knowledge that cervical cancer is caused by HPV. In addition, our research supports the findings of previous literature showing that knowledge of the relationship between HPV infection and penile, oral, and anal cancers is very low: fewer than 20% of our sample knew about these relationships [13]. This low number may indicate that even people who have some knowledge about HPV and who search online for more information still lack information about HPV infection and its relationship to multiple cancers. Greater efforts are needed to inform people that HPV can cause a variety of cancers, and that HPV vaccination is an effective method of preventing these cancers.

Other control variables, such as age, income, occupational status, and English proficiency, were associated with HPV vaccination or HPV vaccination intention. The results of this study confirmed previous findings about factors associated with HPV vaccination [11,29,30].

### Limitations

Although this study indicated that social media usage has a significant role in HPV-related behaviors, this study has some limitations. First, this study was based on secondary data from a public survey. The authors did not develop a survey that focused on specific knowledge about HPV, and the questions about social media usage did not directly address HPV. This limits generalization of our findings about the association between HPV behaviors and social media usage. Second, this study did not examine HPV vaccination intention or vaccine uptake. Although HPV vaccination intention may be highly related to HPV vaccine uptake, some people who initiate HPV vaccination do not complete the entire series of two or three shots required for effective HPV vaccination [31]. Additional research is needed to determine the percentage of people who have HPV vaccination intention and fully complete HPV vaccination. A third limitation of this study is that the respondents were mainly adults. HPV vaccination is suggested for youth and young adults aged 11 to 26 years, [32] who, as a group, are more likely to access and use social media compared to older adults. Further studies that target this group are recommended.

### Conclusions and Implications

Previous research has revealed the significance of HPV awareness, but it has not addressed HPV vaccine awareness and HPV-related knowledge. This study provided further evidence of the nonsignificant relationship between social media usage and HPV-related behaviors. Earlier research has shown that the majority of HPV-related videos and most of the top viewer comments on YouTube reflect antivaccination bias [21]. Our results suggest that there is a lack of high-quality, accurate information on social media. Unlike traditional media, it is hard for health care professionals to intervene through social media. Rather than rely on individual health care workers, government-level policies or efforts are needed to provide accurate information and promote HPV vaccination. Information about HPV has to be accurate and easy for nonprofessionals to understand. A previous study that analyzed Instagram posts about HPV reported that personal stories were the prevalent source of antivaccine postings [33]. Efforts to use storytelling on social media could be one approach to persuade the public. In addition, information that is well suited to social media (eg, infographics) could also increase knowledge about HPV vaccination. An intervention study that used infographics on social media reported that infographics were able to reduce misperceptions about COVID-19 [34]. Government agencies that wish to inform the public with accurate information about HPV should develop communication methods that are appropriate to be shared within social media, based on the public’s level of understanding, and should make strong efforts to disseminate this information within social media.

## Abbreviations

HINTS: Health Information National Trends Survey
HPV: human papillomavirus
OR: odds ratio
